# Inositol (1,4,5)-trisphosphate 5-phosphatase promotes survival of uveal melanoma by regulating oncogenic G protein–driven calcium oscillations

**DOI:** 10.1016/j.jbc.2025.110589

**Published:** 2025-08-12

**Authors:** Michael D. Onken, Carol M. Makepeace, Kevin M. Kaltenbronn, McKenzie Demourelle-Washington, Kisha D. Piggott, Dennis Goldfarb, David J. Kast, Silvia Jansen, Kendall J. Blumer

**Affiliations:** 1 Department of Biochemistry and Molecular Biophysics, Washington University in St Louis, St Louis, Missouri, USA; 2 Department of Cell Biology and Physiology, Washington University in St Louis, St Louis, Missouri, USA; 3 Department of Ophthalmology and Visual Sciences, Washington University in St Louis, St Louis, Missouri, USA

**Keywords:** oncogenic G-proteins, inositol metabolism, calcium currents, palmitoylation, uveal melanoma

## Abstract

Mutant constitutively active (CA) G protein α-subunits encoded by *GNAQ* or *GNA11* (CA-GNAQ/11) drive uveal melanoma (UM), occur in uncommon subtypes of other cancers, and cause Sturge–Weber syndrome and other capillary malformations. CA-GNAQ/11 activates phospholipase Cβ, which cleaves phosphatidylinositol (4,5)-bisphosphate at high rate to produce diacylglycerol that drives oncogenesis and inositol (1,4,5)-trisphosphate (IP3) that releases Ca^2+^ from intracellular stores and triggers store-operated Ca^2+^ entry. For poorly understood reasons, high IP3 flux in UM cells does not elicit Ca^2+^ overload and death. To address this question, we studied INPP5A, a farnesylated, membrane-bound inositol polyphosphate 5-phosphatase that degrades IP3. We showed that INPP5A is upregulated in and required by CA-GNAQ/11-driven UM cell lines and is genetically preserved in UM tumors. We found that INPP5A is reversibly palmitoylated, which together with farnesylation targets the enzyme to subcellular compartments and regulates Ca^2+^ mobilization. Although CA-GNAQ/11 is constitutively active, we discovered that it drives low-frequency Ca^2+^ oscillations in UM cells. We found that acute inhibition of INPP5A in UM cells augments Ca^2+^ oscillation rate, a diagnostic effect of elevating IP3 levels. These results indicated that INPP5A safeguards CA-GNAQ/11-driven UM tumors against Ca^2+^ overload and death by regulating IP3-evoked Ca^2+^ oscillations. As universal frequency–encoded signals, Ca^2+^ oscillations likely regulate vital functions in UM cells. Our findings suggest strategies for targeting INPP5A in diseases or disorders driven by CA-GNAQ/11.

Mutations that constitutively activate (CA) G protein α-subunits encoded by *GNAQ* or *GNA11* (CA-GNAQ/11) drive oncogenesis in >90% of uveal melanoma (UM) tumors (1, 2, 3, 4), occur in uncommon subsets of several other cancers, including ∼10% of cutaneous melanomas and 1% to 5% of other tumors, including glioblastoma multiforme as well as lung, colorectal, and pancreatic adenocarcinomas (5, 6), and cause Sturge–Weber syndrome (7). Although effective therapies for UM and other CA-GNAQ/11-driven diseases and disorders have yet to be developed (3, 8), new therapeutic strategies are emerging. For example, preclinical studies showing that UM cell lines and tumor cells from primary and metastatic UM patients can be arrested by the GNAQ/11 inhibitors, FR900359 (FR) or YM-254890 (9, 10, 11, 12, 13), have led to clinical trials of metastatic UM patients treated with an FR–antibody conjugate (DYP688) directed against a melanocyte cell surface marker (14).

Vulnerabilities caused by CA-GNAQ/11 signaling could provide additional targets for therapeutic investigation. One potential vulnerability is production of inositol (1,4,5)-trisphosphate (IP3) at high flux (9, 15) because of cleavage of phosphatidylinositol (4,5)-bisphosphate (PIP2) by phospholipase Cβ, a ubiquitous downstream effector of CA-GNAQ/11. Because IP3 releases Ca^2+^ from intracellular stores and triggers Ca^2+^ influx *via* store-operated Ca^2+^ entry (SOCE), high IP3 flux could, if unchecked, cause Ca^2+^ overload and death. However, this does not occur in CA-GNAQ/11-driven UM cells (16), suggesting the existence of mechanisms that safeguard against IP3-evoked Ca^2+^ overload and death. One such mechanism is provided by INPP5A (16), a membrane-bound inositol polyphosphate 5-phosphatase (17, 18, 19). INPP5A dephosphorylates IP3 and inositol (1,3,4,5)-tetrakisphosphate (17, 19, 20), producing, respectively, the inactive metabolites, inositol (1,4)-bisphosphate and inositol (1,3,4)-trisphosphate. Indeed, INPP5A has been shown by knockdown studies to protect CA-GNAQ/11-driven UM cell lines from Ca^2+^ overload and death, thereby promoting growth and survival of xenografted UM tumors (16). Similarly, INPP5A knockout causes degeneration of cerebellar Purkinje cells (21), a process dependent on augmented Ca^2+^ flux (22). Additional mechanisms defined in other systems such as IP3 receptor desensitization or downregulation (23, 24, 25) might also safeguard UM cells against Ca^2+^ overload.

The effects of depleting INPP5A in UM cell lines and xenografted tumors raised several important questions that are the focus of studies reported herein. Do tumors from UM patients require INPP5A? Does INPP5A localize to subcellular compartments involved in Ca^2+^ mobilization? What mechanisms are responsible for subcellular targeting and function of INPP5A? Does the level of INPP5A expressed in CA-GNAQ/11-driven UM cells permit or largely suppress IP3-induced Ca^2+^ mobilization?

## Results

### INPP5A is required by and upregulated in CA-GNAQ/11-driven UM cells

**Figure 1:**
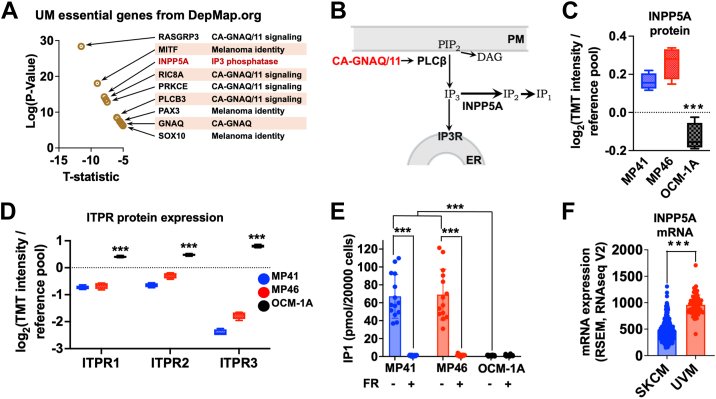
**INPP5A is essential for survival in UM cells driven by CA-GNAQ/11.***A*, genes required by UM cell lines in RNA interference and CRISPR–guide RNA screens ranked by significance and T-statistic. *INPP5A* was the third most highly ranked gene. *B*, schematic of IP3 signaling downstream of constitutively active CA-GNAQ/11. Phospholipase C-beta (PLCβ) activated by CA-GNAQ/11 cleaves phosphatidylinositol (4,5)-bisphosphate (PIP2) at the plasma membrane (PM) to produce diacylglycerol (DAG) and inositol trisphosphate (IP3). IP3 binds IP3 receptors (IP3Rs) on the endoplasmic reticulum (ER) to release Ca^2+^ stores into the cytoplasm. INPP5A regulates Ca^2+^ release by dephosphorylating IP3 to IP2, an inactive metabolite that is further degraded to IP1. *C* and *D*, relative protein abundance calculated from proteomics data. Abundance of INPP5A (*C*) and IP3R isoforms (*D*) in MP41 (GNA11-Q209L), MP46 (GNAQ-Q209L), and OCM-1A (BRAF-V600E) cells are indicated (∗∗∗*p* < 0.001). *E*, IP1 levels in UM cell lines treated with vehicle or FR (∗∗∗*p* < 0.001). *F*, gene expression data from The Cancer Genome Atlas (TCGA). INPP5A mRNA levels in skin cutaneous melanoma (SKCM) and UM (UVM) tumors are indicated (∗∗∗*p* < 0.001). CA, constitutively activate; FR, FR900359; UM, uveal melanoma.

We determined that UM cell lines require INPP5A by querying the DepMap portal (https://depmap.org/portal), which collates data from high-throughput, genome-wide loss-of-function RNA interference and CRISPR–guide RNA screens of cell lines derived from many cancers and diseases (26). Our analysis indicated that *INPP5A* belongs to a group of genes whose depletion significantly impairs growth or survival of CA-GNAQ/11-driven UM cell lines. *INPP5A* ranked third most significantly in this gene set as indicated by unsupervised analysis (Fig. 1*A*). This result was striking because other genes in this group encode proteins required for oncogenic signaling in UM cells (*RASGRP3*, *GNAQ*, *PLCB3*, *PRKCE*, and *RIC8A*; Fig. 1, *A* and *B*) or specification of melanocyte identity (*MITF*, *PAX3*, and *SOX10*). In contrast, our analysis of DepMap data indicated that *INPP5A* is not required significantly by cell lines that express WT GNAQ/11, indicating that *INPP5A* dependence is characteristic of CA-GNAQ/11-expressing cell lines.

In an orthogonal approach, we found that INPP5A is differentially upregulated in UM cell lines driven by CA-GNAQ/11 *versus* constitutively active BRAF (CA-BRAF; BRAF-V600E). We performed quantitative analysis of a comprehensive set of proteomic data from CA-GNAQ/11-driven (MP41 and MP46) and CA-BRAF-driven (OCM-1A) UM cell lines (27). This analysis indicated that INPP5A protein levels are 1.3-fold higher (*p* < 0.001) in UM cells driven by CA-GNAQ/11 than in CA-BRAF-driven UM cells (Fig. 1*C*).

Further analysis of this proteomic dataset indicated that all three IP3 receptor subtypes are underexpressed twofold (ITPR1 and 2) to eightfold (ITPR3) (*p* < 0.001) in CA-GNAQ/11-driven UM cells relative to CA-BRAF-driven UM cells (Fig. 1*D*). This result suggested that IP3 flux is greater in CA-GNAQ/11-driven UM cell lines because IP3 receptors undergo ubiquitin-mediated proteolysis in response to IP3 binding (28). Indeed, we found that levels of IP1, a product of IP3 turnover that accumulates in the presence of LiCl, are >100-fold greater in CA-GNAQ/11-driven UM cells than in CA-BRAF-driven UM cells (Fig. 1*E*). This difference in IP1 level depended on CA-GNAQ/11 signaling because it was eliminated by the GNAQ/11 inhibitor FR (Fig. 1*E*). These results suggested that UM cell lines driven by CA-GNAQ/11 adapt to the stress of high IP3 flux in part by upregulating INPP5A protein levels and downregulating IP3 receptors.

We subsequently determined that tumors from UM patients upregulate and require INPP5A by comparing nonredundant, curated sets of tumor transcriptomic and genomic data (cBioPortal) from UM (80 samples) and skin cutaneous melanoma (SKCM; 488 samples) patients. We found that *INPP5A* mRNA expression is significantly (*p* < 0.001) higher in UM tumors compared with SKCM tumors (Fig. 1*F*) and that *INPP5A* is genetically preserved in UM tumors compared with SKCM tumors (χ^2^ < 0.001) as indicated by analysis of tumor genomic data. *INPP5A* deep deletions (presumptive homozygous deletions) and nonsense mutations were lacking in all UM tumors in the database. Only one *INPP5A* mutation occurred in a UM tumor, which encoded a Q412L substitution at the C terminus, distal to the catalytic domain. Whereas this substitution would not disrupt enzyme activity, it would switch the isoprenoid lipid attached to INPP5A for membrane targeting from a farnesyl to a geranylgeranyl moiety (29), which has the potential of affecting diffusional mobility on membranes (30). In contrast, *INPP5A* mutations were present in SKCM tumors, including deep deletions (1.8%) and missense or nonsense mutations (2.5%). This evidence indicated that *INPP5A* is genetically inactivated in SKCM but not UM tumors.

### INPP5A localizes to subcellular compartments involved in Ca^2+^ mobilization

**Figure 2:**
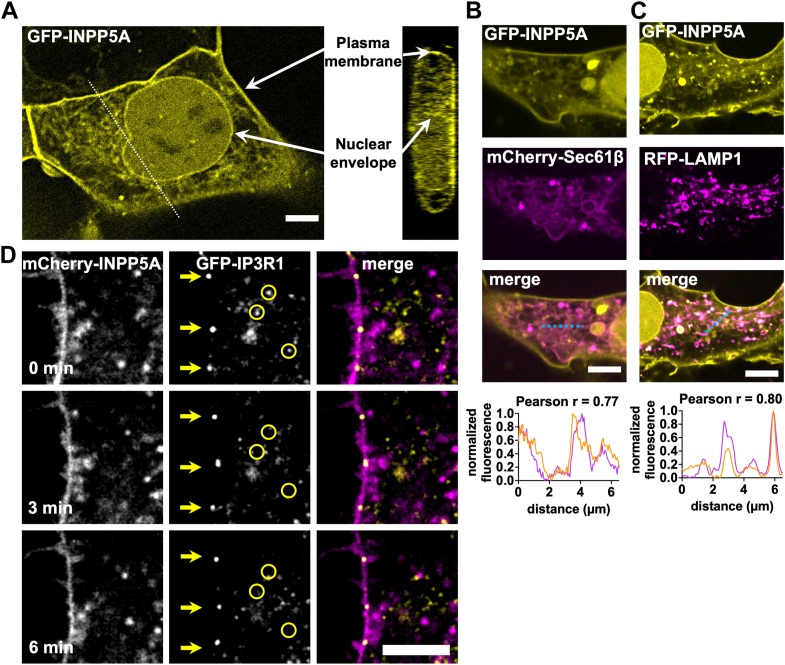
**INPP5A localizes to subcellular compartments including immobile IP3 receptor clusters that mediate store-operated Ca^2+^ entry.** HEK293 cells stably expressing GFP-INPP5A were imaged on a spinning-disk confocal fluorescent microscope. *A*, GFP-INPP5A localized to the plasma membrane and nuclear envelope (*arrow*) and other subcellular compartments. Scale bar represents 5 μm. *B*, colocalization of GFP-INPP5A (*top panel*) and the ER marker mCherry-SEC61B (*middle panel*) indicated by overlap of the two fluorescent signals (*bottom panel*). Scale bar represents 5 μm. Graph at the *bottom* shows line scans (indicated by a *dotted line*) of normalized GFP-INPP5A and mCherry-SEC61B fluorescence intensities. Pearson's correlations were averaged from 10 independent line scans. *C*, colocalization of GFP-INPP5A (*left panel*) and the lysosomal marker RFP-LAMP1 (*middle panel*) indicated by overlap of the two fluorescent signals (*right panel*). Scale bar represents 5 μm. Graph at the *bottom* shows line scans (indicated by a *dotted line*) of normalized GFP-INPP5A and RFP-LAMP1 fluorescence intensities. Pearson's correlations were averaged from 10 independent line scans. *D*, previous studies (32) have shown that highly stable IP3R1 clusters adjacent to the plasma membrane are responsible for calcium signaling in cells. Coincidence of plasma membrane–localized mCherry-INPP5A (*left panel*) and immobile IP3 receptor (EGFP-ITPR1, *middle panel*) clusters at junctions between the plasma membrane and ER was examined in HeLa cells. Coincidence of the two fluorescence signals (*right panel*) was indicated by live-cell fluorescence video microscopy. Initial positions of immobile (*arrows*) and mobile (*circles*) subsets of EGFP-ITPR1 at each time point in the video are indicated. Scale bar represents 5 μm. ER, endoplasmic reticulum; HEK293, humane embryonic kidney 293 cell line; IP3, inositol (1,4,5)-trisphosphate; LAMP1, lysosomal-associated membrane protein 1; RFP, red fluorescent protein.

Determining where INPP5A localizes within cells is important for understanding how this enzyme supports growth and survival of UM cells and tumors. This question remained unanswered because prior studies used transient overexpression to show that C-terminal farnesylation of INPP5A targets the enzyme to undefined subcellular compartments (18). Because endogenously expressed INPP5A was undetectable with available antibodies, we addressed this question by stably expressing GFP-INPP5A with or without organelle marker proteins tagged with mCherry or red fluorescent protein (RFP) in human embryonic kidney 293 (HEK293) cells and performing live-cell confocal fluorescence microscopy. While recognizing limitations potentially posed by stable overexpression, we found that WT GFP-INPP5A localizes to the plasma membrane (PM) and nuclear envelope (Fig. 2*A*, *arrows*) as well as the endoplasmic reticulum (ER; Fig. 2*B*) as indicated by overlap with an ER transmembrane protein, mCherry-tagged Sec61β (mean Pearson’s coefficient *r* = 0.77). GFP-INPP5A also localized to large intracellular puncta/vesicles unlabeled by mCherry-Sec61β. Many of these structures are components of lysosomal pathway, as indicated by colocalization of GFP-INPP5A with a lysosomal-associated membrane protein 1–RFP fusion protein (Fig. 2*C*; mean Pearson’s coefficient *r* = 0.80) (31).

Receptors coupled to GNAQ/11 elicit SOCE that is mediated by an immobile subset of IP3 receptors localized to ER–PM junctions containing STIM–ORAI channels (32). We therefore determined whether INPP5A has the potential of regulating GNAQ/11-driven SOCE by localizing near immobile IP3 receptors. To preclude IP3 receptor mislocalization because of overexpression, we used a published HeLa cell line in which enhanced GFP (EGFP) is fused to IP3 receptor R1 expressed from its chromosomal locus (32) as the recipient for stably expressing mCherry-INPP5A by lentiviral transduction. Live-cell dual-color fluorescence single-slice confocal video microscopy (Fig. 2*E* and Movie S1) distinguished immobile clusters of EGFP-IP3R1 near the PM (initial positions marked by *arrows*) from the mobile subpopulation of EGFP-IP3R1 on regions of the ER distal to the PM (initial positions marked by *circles*). This analysis indicated that PM–bound mCherry-INPP5A overlapped with immobile clusters of EGFP-IP3R1, whereas little overlap was observed between mCherry-INPP5A and the mobile subset of EGFP-IP3R1 clusters on the ER. These results indicated that INPP5A is poised to regulate IP3 turnover near immobile IP3 receptor clusters that mediate SOCE.

### Subcellular targeting of INPP5A by farnesylation and palmitoylation

**Figure 3:**
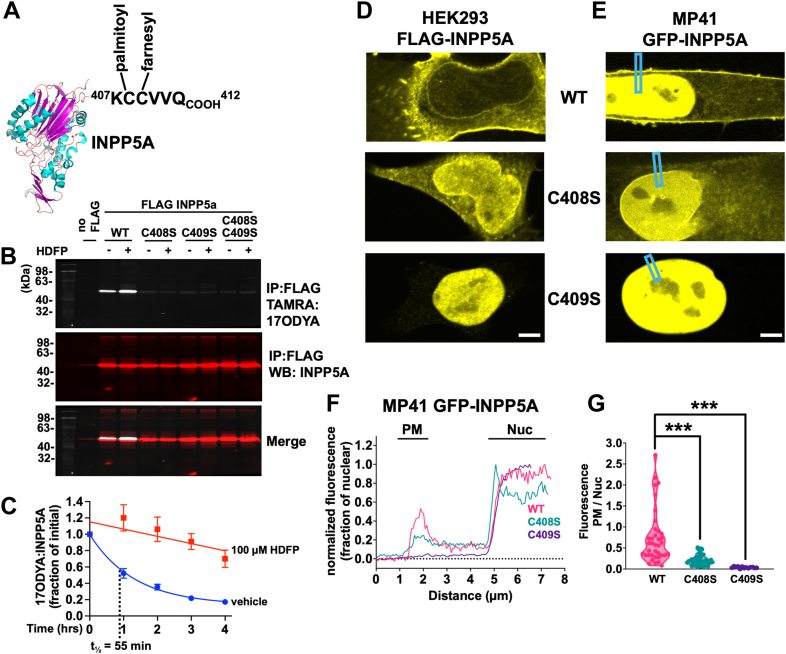
**Palmitoylation of INPP5A.***A*, cysteine-408 of INPP5A is a potential palmitoylation site. The C terminus of INPP5A (AlphaFold structural model shown) includes a farnesylation site (CVVQ) preceded by cysteine-408, a potential palmitoylation site. *B*, palmitoylation of INPP5A. Palmitoylation was detected by labeling HEK293 cells expressing FLAG-INPP5A with the palmitate analog 17-octadecynoic acid (17-ODYA). 17-ODYA attached to immunoprecipitated FLAG-INPP5A was detected by using click chemistry to conjugate the TAMRA fluorophore to the palmitate analog. Results shown were obtained with HEK293 cells expressing WT FLAG-INPP5A (WT) and mutants lacking the palmitoylation site (C408S), the farnesylation site (C409S), or both (C408S/C409S). *C*, palmitate turnover on INPP5A. Turnover was measured with a 17ODYA/palmitate pulse-chase followed by immunoprecipitation, TAMRA conjugation, fluorescence detection, and immunoblotting. *D*, membrane targeting of INPP5A requires palmitoylation and farnesylation of the enzyme. Subcellular localization of the indicated palmitoylation and farnesylation site mutants of 3XFLAG-INPP5A were confirmed by confocal microscopy of fixed HEK293 cells. Scale bar represents 5 μm. *E*, subcellular localization of the indicated palmitoylation and farnesylation site mutants of GFP-INPP5A was further confirmed by live-cell confocal microscopy of MP41 cells. Scale bar represents 5 μm. *F*, line scans (indicated by *boxed rectangles* in *E*) of GFP-INPP5A fluorescence intensities including the plasma membrane (PM) and nucleus (nuc) in MP41 cells were normalized to peak nuclear fluorescence. Representative scans are shown in the *left panel* for WT, C408S palmitoylation–deficient mutants, and C409S farnesylation–deficient mutants. *G*, PM fluorescence intensities normalized to mean nuclear intensities from 35 independent scans for each genotype are plotted in the *right panel*. ∗∗∗*p* < 0.001. HEK293, human embryonic kidney 293 cell line.

We noticed that INPP5A possesses a candidate palmitoylation site (C408; Fig. 3*A*) immediately preceding its known C-terminal farnesylation site (C409 (18)). This observation was of interest because palmitate attachment by thioester linkage to cysteine residues adjacent to farnesylation sites often facilitates protein subcellular localization and function (33). Furthermore, palmitoylation regulates function of many proteins because often it is dynamic and enzymatically reversible (34, 35).

We tested this hypothesis by demonstrating that INPP5A is palmitoylated on C408. Palmitoylation was detected by using the palmitate analog 17-ODYA to label HEK293 cells stably expressing WT or mutant forms of FLAG-INPP5A in which the putative palmitoylation (C408S) and/or farnesylation (C409S) sites were mutated. 17-ODYA attached to FLAG-INPP5A following immunoprecipitation was detected by fluorophore (TAMRA) conjugation with click chemistry. We found that 17-ODYA labeled WT FLAG-INPP5A but not the C408S, C409S, or C408S/C409S mutants (Fig. 3*B*). These results indicated that C408 is palmitoylated by a mechanism dependent on farnesylation, as occurs in other proteins (33). We determined whether FLAG-INPP5A palmitoylation is static or dynamic by treating 17-ODYA-labeled cells with hexadecylfluorophosphonate (HDFP (36)), an inhibitor of acylprotein thioesterases that depalmitoylate proteins (35). Results indicated that HDFP increases steady-state 17-ODYA labeling of FLAG-INPP5A ∼2-fold (Fig. 3*B*), suggesting that FLAG-INPP5A is reversibly rather than statically palmitoylated. We tested this hypothesis by measuring palmitate turnover on FLAG-INPP5A in a 17-ODYA/palmitate pulse-chase experiment. Results indicated that palmitate turnover occurs on FLAG-INPP5A with a half time of ∼55 min (Fig. 3*C*; range: 40–81 min determined by single-phase decay regression analysis), similar to other proteins whose subcellular trafficking and function are dynamically regulated by palmitate turnover (34).

We found that palmitoylation facilitates subcellular targeting of INPP5A, as indicated by immunofluorescence and live-cell imaging (Fig. 3, *D*–*G*). Mutation of the palmitoylation site (C408S) reduced localization of FLAG- and GFP-INPP5A to the PM and ER and increased association with the nuclear envelope in HEK293 cells (Fig. 3*D*) and in a UM cell line (MP41) (Fig. 3*E*). Mutation of the farnesylation site (C409S) prevented association of tagged INPP5A with cellular membranes and caused accumulation in the nucleoplasm in both cell lines (Fig. 3, *D* and *E*). Line scan analysis was used to quantify localization of WT and mutant GFP-INPP5A to the PM in MP41 cells (n = 35 cells/condition). We found that nonpalmitoylated GFP-INPP5A (C408S) exhibited significant (*p* < 0.001) reduction in PM localization (Fig. 3*E*), and nonfarnesylated GFP-INPP5A (C409S) failed to associate detectably with the PM (Fig. 3*E*). Palmitoylation and farnesylation therefore are essential for proper subcellular targeting of INPP5A.

### CA-GNAQ/11 drives Ca^2+^ oscillations in UM cells

Whether upregulated INPP5A in CA-GNAQ/11-driven UM cells prevents or simply regulates IP3-evoked Ca^2+^ mobilization in UM cells is unknown because, to our knowledge, Ca^2+^ dynamics in UM cells had not been studied before with appropriate temporal resolution. We addressed this question by using live-cell fluorescence video microscopy to measure single-cell Ca^2+^ dynamics in UM cell lines driven by CA-GNAQ/11 (MP41, MP46) *versus* BRAF (OCM-1A) as indicated by Oregon Green 488-BAPTA-1, AM (OGB1), a cell-permeable fluorescent dye that increases fluorescence emission upon Ca^2+^ binding.

**Figure 4:**
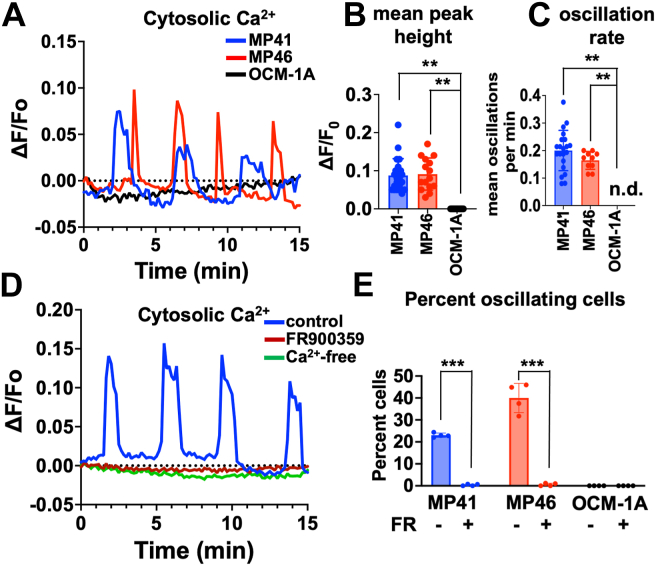
**CA-GNAQ/11-driven UM cells exhibit Ca^2+^ oscillations.** MP41 (GNA11-Q209L), MP46 (GNAQ-Q209L), and OCM-1A (BRAF-V600E) UM cells were loaded with Oregon Green 488-BAPTA, and fluorescence emission was measured over time to detect changes in cytoplasmic Ca^2+^ levels. *A*, representative Ca^2+^ traces for individual cells from the indicated UM cell lines. *B*, maximum height of each Ca^2+^ transient was determined, and mean peak heights were calculated for individual cells. *C*, Ca^2+^ oscillation periodicity (frequency) was measured as the time between the start of one transient until the start of the subsequent transient for each individual cell trace and expressed as oscillations per minute. *D*, representative Ca^2+^ traces for individual MP46 cells treated with vehicle or FR for 24 h, or placed in Ca^2+^-free medium for 1 h. *E*, fraction of cells per field that exhibited multiple transients were calculated for each cell line treated with vehicle or FR for 24 h. For all panels, ∗∗*p* < 0.01; ∗∗∗*p* < 0.001. CA, constitutively activate; FR, FR900359; UM, uveal melanoma.

Results indicated that CA-GNAQ/11-driven UM cell lines exhibit spontaneous low frequency (∼0.2/min), baseline-separated Ca^2+^ oscillations (Fig. 4*A* and Movies S2 and S3). This result was unexpected because CA-GNAQ expressed ectopically in cardiomyocytes had been shown to drive Ca^2+^ oscillations with much higher frequency (∼5/min) (37). Ca^2+^ oscillations in two CA-GNAQ/11-driven UM cell lines exhibited similar mean values for peak heights and oscillation frequencies but differed somewhat in terms of maximal values of peak height (Fig. 4*B*) and frequency (Fig. 4*C*) and average percentage of oscillating cells (Fig. 4*E*). These Ca^2+^ oscillations are driven by CA-GNAQ/11 signaling because they were abolished by the GNAQ/11 inhibitor FR (Fig. 4, *D* and *E* and Movie S4), and they did not occur in CA-BRAF-driven UM cells (Fig. 4, *A*, *B*, *C*, and *E* and Movie S5). When CA-GNAQ/11-driven UM cells were analyzed in the absence of extracellular Ca^2+^ (Fig. 4*D* and Movie S6), Ca^2+^ oscillations were not observed, suggesting that Ca^2+^ entry is required. Thus, rather than completely blocking Ca^2+^ mobilization, upregulated INPP5A in CA-GNAQ/11-driven UM cell lines allows IP3-driven Ca^2+^ mobilization to occur.

**Figure 5:**
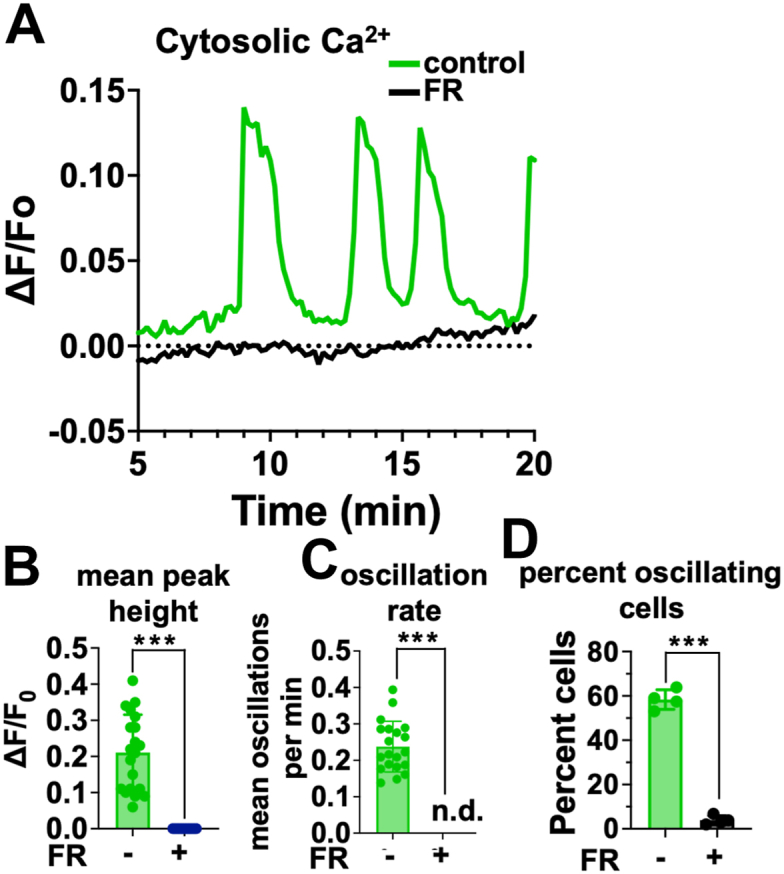
**Primary human UM cells exhibit Ca^2+^ oscillations.***A*, representative Ca^2+^ traces for individual cells from a primary human UM tumor sample treated with vehicle or FR for 24 h. *B*, mean peak heights were calculated for primary UM tumor cells treated with vehicle or FR as in (*B*). *C*, oscillation periodicity (frequency) was calculated for primary UM tumor cells treated with vehicle or FR as in (*C*). *D*, fraction of UM tumor cells per field that exhibited multiple Ca^2+^ transients when treated 24 h with vehicle or FR. ∗∗∗*p* < 0.001. FR, FR900359; UM, uveal melanoma.

We also observed Ca^2+^ oscillations in primary UM tumor cells from a patient biopsy. Replicate measurements were made by dividing the sample and establishing short-term cultures that enriched for adherent tumor cells. We found that tumor cells from this UM patient exhibit spontaneous low-frequency, baseline-separated Ca^2+^ oscillations similar to those observed in UM cell lines (Fig. 5*A* and Movie S7). These Ca^2+^ oscillations are driven by CA-GNAQ/11 because they were abrogated by FR (Fig. 5, *A*–*D* and Movie S8). Their frequencies were similar to those observed in UM cell lines (compare Figs. 4*C* and 5*C*). However, peak heights and the percentage of oscillating cells were greater than observed in UM cell lines (compare Figs. 4*B* and 5*B*; *p* < 0.01; and Figs. 4*E* and 5*D*; *p* < 0.01). Results obtained with this UM tumor sample and UM cell lines therefore suggest that Ca^2+^ oscillations are a common feature of UM cells driven by CA-GNAQ/11.

### INPP5A regulates Ca^2+^ oscillations in UM cells

Ca^2+^ oscillations in UM cell lines were not reported in a prior study examining the effects of INPP5A knockdown (16). In those experiments, the temporal resolution of Ca^2+^ imaging was low (hours to days), INPP5A depletion was slow, and cells died as steady-state Ca^2+^ levels increased. Accordingly, to determine whether INPP5A regulates Ca^2+^ oscillations in UM cells, we hypothesized it would be necessary to acutely inhibit the enzyme and perform live-cell Ca^2+^ imaging with greater temporal resolution (seconds to minutes). Accordingly, we used the only inhibitor currently available that targets INPP5A, YU144369 (YU) (38). Whereas YU also inhibits other 5-phosphatases (INPP5B, INPP5E, SHIP1, Synaptojanin 1, Synaptojanin 2, and OCRL), these enzymes preferentially dephosphorylate phosphoinositide lipids (39) and thus were unlikely to affect Ca^2+^ dynamics during the short time frame of our experiments.

**Figure 6:**
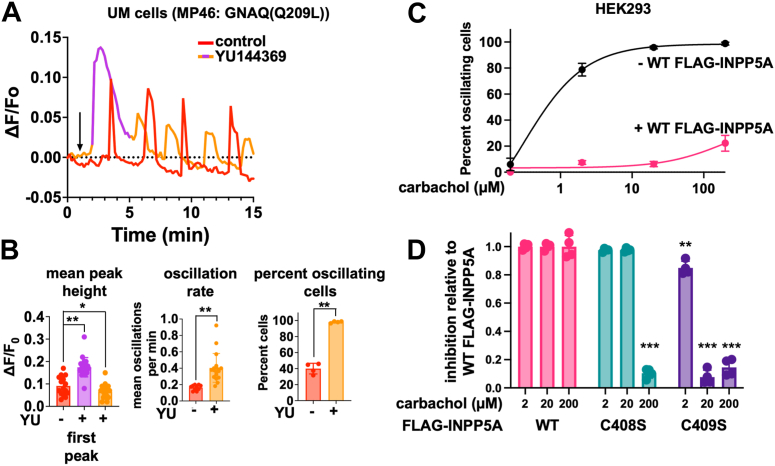
**Membrane-localized INPP5A regulates Ca^2+^ oscillations in cells.***A*, representative Ca^2+^ traces for individual MP46 cells after vehicle or YU144369 (YU; 100 μM) was applied 1 min (*arrow*) after image imaging was started. *B*, mean peak height of the first Ca^2+^ transient observed after YU injection was calculated (*left panel*), oscillation periodicity (frequency) was measured after the first peak in YU-treated cells (*center panel*), and percent of cells per field that exhibited multiple Ca^2+^ transients after the first peak were calculated for vehicle- and YU-treated cells (*right panel*). For all panels, ∗*p* < 0.05; ∗∗*p* < 0.01. *C*, HEK293 cells were stimulated with indicated dosages of carbachol to induce Gq-driven Ca^2+^ transients through activation of muscarinic cholinergic receptors. Percents of cells per field that exhibited multiple Ca^2+^ transients were calculated in 3XFLAG-INPP5A expressing (+) or control (−) cells. *D*, carbachol dose responses were carried out as in *C*) in HEK293 cells expressing WT 3XFLAG-INPP5A (WT) or the palmitoylation mutant (C408S) or the farnesylation mutant (C409S). Inhibition was calculated as fraction of inhibition relative to WT; ∗∗*p* < 0.01; ∗∗∗*p* < 0.001. HEK293, human embryonic kidney 293 cell line.

We analyzed the effects of INPP5A inhibition on Ca^2+^ oscillations in CA-GNAQ-driven UM cells (MP46) by adding YU or vehicle to the medium 1 min after imaging was started (Fig. 6*A*, *arrow*, and Movie S9) and recording images at 10 s intervals. Results indicated that YU increases the magnitude of the first Ca^2+^ spike (Fig. 6*A*, *purple trace*). After this initial spike, subsequent Ca^2+^ spikes (Fig. 6*A*) in YU-treated UM cells had smaller peak heights but occurred more frequently and in a greater proportion of cells than in vehicle controls (Fig. 6*B*). Because higher Ca^2+^ oscillation frequency is diagnostic of increased IP3 levels (40, 41), these results indicated that YU inhibits INPP5A to augment IP3 levels. Furthermore, because Ca^2+^ oscillations universally regulate many vital cell biological processes (42, 43, 44), these results in conjunction with prior knockdown studies indicated that upregulated INPP5A has two functions in UM tumors: (1) preventing IP3-induced Ca^2+^ overload and (2) regulating Ca^2+^ oscillations that likely support important biological functions in UM tumors.

### Functional effects of preventing palmitoylation or prenylation of INPP5A

To determine whether the function of INPP5A requires palmitoylation or prenylation of the enzyme, we stably expressed WT and mutant forms of FLAG-INPP5A in HEK293 cells and measured the proportion of cells exhibiting Ca^2+^ oscillations in response to agonist (carbachol) stimulation of endogenously expressed GNAQ/11-coupled muscarinic acetylcholine receptors. In the absence of FLAG-INPP5A, nearly all HEK293 cells exhibited Ca^2+^ oscillations at every concentration of carbachol examined (Fig. 6*C* Stable overexpression of WT FLAG-INPP5A strongly reduced the proportion of cells exhibiting Ca^2+^ oscillations at every dose of carbachol studied (Fig. 6*C*). In contrast, stable overexpression of nonpalmitoylated (C408S) and nonprenylated (C409S) FLAG-INPP5A was significantly less effective at inhibiting Ca^2+^ oscillations, as indicated by the fraction of cells exhibiting Ca^2+^ oscillations as a function of carbachol concentration (Fig. 6*D*). Nonpalmitoylated FLAG-INPP5A (C408S) strongly inhibited Ca^2+^ oscillations at low and moderate but not high concentration of carbachol (Fig. 6*D*), whereas nonprenylated FLAG-INPP5A strongly inhibited Ca^2+^ oscillations only at the lowest concentration of carbachol studied (Fig. 6*D*). These results in conjunction with imaging data therefore indicated that palmitoylation and prenylation promote subcellular localization and function of INPP5A as a regulator of GNAQ/11-driven Ca^2+^ mobilization.

## Discussion

We have identified signaling and regulatory mechanisms relevant to diseases or disorders in which CA-GNAQ/11 is expressed. Our results demonstrate that the inositol polyphosphate 5-phosphatase INPP5A that degrades IP3 is upregulated in and required by UM cell lines and tumors; palmitoylation and farnesylation of INPP5a promote the function of this enzyme and target it to compartments bearing IP3 receptors that release Ca^2+^ from intracellular stores and trigger Ca^2+^ entry; CA-GNAQ/11 drives low-frequency Ca^2+^ oscillations in UM cells; and overexpressed INPP5A in UM cells regulates rather than blocks CA-GNAQ/11-driven Ca^2+^ oscillations. As discussed later, our findings raise questions regarding the mechanisms by which CA-GNAQ/11 drives Ca^2+^ oscillations in UM cells, the biological functions of these Ca^2+^ oscillations, and approaches to target INPP5A therapeutically.

### Augmenting INPP5A function to safeguard UM cells against Ca^2+^ overload

Two mechanisms potentially augment INPP5A function to safeguard CA-GNAQ/11-driven UM cells against Ca^2+^ overload. INPP5A protein upregulation may provide one mechanism even though this effect is modest (1.3-fold) as indicated by our quantitative proteomic analyses of CA-GNAQ/11- *versus* BRAF-driven UM cells. Activation of INPP5A catalytic activity may provide another mechanism because CA-GNAQ/11 signaling in UM cells activates PKC isoforms (27), and PKC phosphorylation of INPP5A increases *V*_max_ of this phosphatase threefold (45). Thus, the combined effect of these two mechanisms potentially could increase INPP5A function ∼4-fold.

### Ca^2+^ oscillations in UM cells

Ca^2+^ oscillations in many cell types are evoked by agonists that activate receptors coupled to WT GNAQ/11 (44) and can be driven by ectopic expression of CA-GNAQ (37). However, Ca^2+^ oscillations in UM cells are unusual because of their low frequency (∼0.2/min), suggesting that they are elicited or regulated by novel mechanisms. Determining how CA-GNAQ/11 drives low-frequency Ca^2+^ oscillations in UM cells would require the following mechanisms to be addressed. First is whether IP3 production by PLCβ in UM cells is constant or oscillatory, which could depend on whether mechanisms such as PLCβ activation by Ca^2+^ (46, 47) and/or inhibition by protein kinase C phosphorylation (48) are operative. Second is whether Ca^2+^ oscillations in the presence of oscillating (49) or constant (40, 41) IP3 involve mechanisms downstream of PLCβ. Ca^2+^ oscillations can occur when IP3 levels are constant because IP3 receptors are positively and negatively regulated, respectively, by Ca^2+^ and IP3 (40, 41). Ca^2+^ oscillations can occur when IP3 production is constant but IP3 levels oscillate because of Ca^2+^-dependent activation of kinases that convert IP3 to inositol (1,3,4,5)-tetrakisphosphate (50). Third is whether Ca^2+^ oscillations in UM cells occur at low frequency because IP3 consumption is augmented by overexpressed INPP5A. This hypothesis is suggested by our finding that an INPP5A inhibitor increases Ca^2+^ oscillation frequency (Fig. 4*C*), and by IP3 uncaging experiments in other systems showing that an IP3 pulse increases Ca^2+^ spike frequency (40, 41).

### PIP2 resupply

To support Ca^2+^ oscillations and oncogenesis in UM cells, PIP2 cannot be consumed completely by CA-GNAQ/11-driven PLCβ. In contrast, PIP2 can be consumed completely by PLCβ within minutes after agonist activation of receptors coupled to WT GNAQ/11 (46). Accordingly, we hypothesize that UM cells prevent PIP2 depletion by accelerating metabolic turnover and recycling of PIP2 cleavage products, IP3 and diacylglycerol (DAG). Overexpressed INPP5A could facilitate PIP2 resupply because this enzyme dephosphorylates IP3 in pathways leading to production of inositol for PIP2 synthesis (51). Beyond INPP5A, other phosphatases in the inositol regeneration pathway (51) or CDP-DAG synthases that catalyze the rate-limiting step for production of phosphatidylinositol from DAG (52) might be overexpressed or activated to drive PIP2 resupply. Indeed, because CDP-DAG synthases, CDS1 and CDS2, are inhibited by PIP2 (53), these enzymes might be disinhibited in UM cells as PIP2 is hydrolyzed by PLCβ. By facilitating PIP2 resupply, this mechanism could be critical for sustaining Ca^2+^ oscillations and oncogenesis in CA-GNAQ/11-driven UM cells.

### Functions of Ca^2+^ oscillations in UM cells

Ca^2+^ oscillations universally provide frequency-encoded signals that regulate vital cell biological processes (44, 54). Because Ca^2+^ oscillations in UM cells are driven by CA-GNAQ/11, they potentially support a subset of the biological processes known to be driven by these oncogenic G proteins, including proliferation, survival, transcription, mitochondrial metabolism, dedifferentiation, and transendothelial migration (9, 12, 27, 55, 56). Determining which of these biological processes are elicited by Ca^2+^ oscillations will require methods to conditionally inhibit this signaling mechanism without impairing other mechanisms driven by CA-GNAQ/11.

### Targeting INPP5A

INPP5A potentially could be targeted in UM or other CA-GNAQ/11-driven diseases or disorders by small molecules that inhibit its catalytic activity (57), farnesylation (58), or palmitoylation (59). Novel and highly selective small-molecule inhibitors of INPP5A enzyme activity are likely to be required because the currently available inhibitor (YU) also targets 5-phosphatases that dephosphorylate phosphoinositide lipids required for many cell biological processes (38). Furthermore, INPP5A inhibitors delivered selectively to UM tumors probably would be required because systemic delivery could have unacceptable side effects based on evidence that loss of the *INPP5A* gene is associated with development or progression of squamous cell carcinoma (60, 61), glioma (62, 63), and leukemia (61), as well as ataxia and cerebellar degeneration (21, 64). An alternative of targeting INPP5A farnesylation by depleting farnesyl pyrophosphate with statins or inhibiting farnesyltransferase is suggested by evidence, respectively, that cerivastatin enhances efficacy of MEK inhibition toward xenografted UM tumors (65) and that tipifarnib or lonafarnib induces apoptosis of CA-GNAQ/11-driven UM cell lines (66). UM tumors might respond durably to farnesyltransferase inhibition because the C-terminal glutamine residue of the INPP5A CAAX box precludes alternative prenylation by geranylgeranyltransferase I (67). In contrast, K- or N-Ras-driven tumors respond nondurably to farnesyltransferase inhibition because these oncoproteins can be alternatively geranylgeranylated (68, 69).

In conclusion, mechanisms revealed by our studies suggest new opportunities for developing strategies to treat metastatic UM, an aggressive and rapidly fatal disease. Mechanistic principles and therapeutic strategies emerging from studies of UM may apply to other poorly treated tumors, diseases, or disorders in which CA-GNAQ/11 is expressed.

## Experimental procedures

### Cell lines and reagents

FR was purified from *Ardisia crenata* according to the published methods (70). The human UM PDX-derived cell lines MP41 (American Type Culture Collection [ATCC]; catalog no.: CRL-3297, Research Resource Identifier [RRID]: CVCL_4D12) and MP46 (ATCC; catalog no.: CRL-3298, RRID: CVCL_4D13) (55) were purchased from ATCC. The human UM cell line OCM-1A (RRID: CVCL_6934) was derived by and was the generous gift of Dr June Kan-Mitchell (Biological Sciences, University of Texas at El Paso). HeLa cells expressing EGFP-IP3R1 fusion protein from the endogenous ITPR1 locus (32) were the generous gift of Dr David Yule (Department Pharmacology and Physiology, University of Rochester). All cell lines were grown at 37 °C in 5% CO_2_ in RPMI1640 medium (Life Technologies) supplemented with antibiotics and 25% fetal bovine serum (FBS) (MP41 and MP46) or 10% FBS (OCM-1A). Cells were not grown above passage 35. DNA sequences encoding GFP-INPP5A and 3XFLAG-INPP5A were inserted into the pCDH-CMV-MCS-EF1-Puro lentivector expression plasmid, and lentiviral particles were produced as described previously (55). Cells were incubated with lentivirus in a growth medium containing 10 μg/ml protamine sulfate for 24 h before changing to fresh growth medium. At 3 days postinfection, cells were moved to a growth medium containing 1.5 μg/ml puromycin to select for stably integrated cells. TransIT-LT2 transfection reagent (Mirus Bio) was used for all transient transfections according to the manufacturers’ protocol. Lysosomal-associated membrane protein 1-RFP (Addgene #1817) and mCherry-SEC61B (Addgene #90994) expression constructs were used.

### Patients and sample collection

Human tissue samples were obtained with patient-written informed consent and approval of the institutional review board of Washington University in St Louis and adhered to the tenets of the Declaration of Helsinki. Fine-needle aspiration biopsies of primary UM tumors were performed as part of standard of care, which also collected biopsies for cytological evaluation of tumor cells and molecular classification (Castle Biosciences). All samples were collected directly into 2 ml of growth medium in the operating room before being transported to the laboratory. Biopsy samples were centrifuged and resuspended in 500 μl growth medium and then equally divided onto fibronectin-coated MatTek glass-bottom dishes (P35G-1.5-14-C) and grown overnight in a 5% CO_2_ thermal conductivity incubator. Growth medium for primary UM samples is MDMF medium, which consists of HAM’s F12 (Lonza) supplemented with 1 mg/ml bovine serum albumin (Sigma–Aldrich), 2 mM L-glutamine (Lonza), 1X SITE (Sigma–Aldrich), 1× B27 (Gibco), 20 ng/ml basic fibroblast growth factor (PeproTech, Inc), and 50 μg/ml gentamicin (Sigma–Aldrich). Cells were allowed to attach to the substrate before 2 ml of fresh medium was added to each dish.

### IP1 assays

Accumulation of IP1 in UM cells was measured using the IP-One kit (CiSbio, Inc; catalog no.: 62IPAPEB) according to the supplier’s instructions. Cells (20,000 MP41 cells, 20,000 MP46 cells, and 20,000 OCM-1A cells) were seeded into white-bottom tissue culture grade 384-well plates. Following an overnight incubation, cells were treated with FR or vehicle (dimethyl sulfoxide) and returned to the incubator. The next day, stimulation buffer was added for 1 h, after which IP1-d2 and Ab-Cryp were added, and the cells were incubated at room temperature for 60 min. Plates were read in a Synergy H4 plate reader (BioTek). Standard curves were generated using reagents supplied with the kit.

### Protein quantification and statistical analysis

Processed tandem mass tag (TMT)-labeled proteomics data were downloaded from the PRIDE repository (accession no.: PXD038115) (22). The dataset was originally searched using MaxQuant (Max Planck Institute of Biochemistry; http://www.maxquant.org), version 2.0.3.0 and filtered to a 1% false discovery rate at both the peptide and protein levels. To enable comparison of protein abundances across different cell lines, which were distributed across separate TMT experiments, we relied on the presence of two pooled reference channels included in each TMT set. Protein intensities were normalized within each TMT experiment to the average of the two pooled reference samples, allowing comparison of protein abundance across cell lines and conditions. Statistical analysis was performed using Perseus (Max Planck Institute of Biochemistry; http://www.maxquant.org), version 2.0.7.0. For each treatment condition (FR and dimethyl sulfoxide), a separate one-way ANOVA was performed to identify proteins with differential abundance across the three cell lines. *p* Values were adjusted using the Benjamini–Hochberg procedure to control the false discovery rate. For proteins with significant ANOVA results, Tukey’s *post hoc* test was used to identify specific pairwise differences between cell lines.

### Ca^2+^ imaging

Cells were plated on MatTek dishes with coverglass bottoms coated with fibronectin and grown overnight at 37 °C. Cells were rinsed in Dulbecco’s PBS (DPBS) at room temperature and then loaded with Oregon Green 488-BAPTA-1, AM in FluoroBrite Dulbecco's modified Eagle's medium (DMEM) containing pluronic F-127 detergent for 1 h at room temperature. Staining solution was removed, and cells were rinsed twice with DPBS at room temperature to remove extracellular dye and then placed in 1 ml FluoroBrite DMEM for imaging at room temperature. For Ca^2+^-free experiments, cells were imaged in 1 ml DPBS supplemented with GlutaMAX and sodium pyruvate. For drug treatment experiments, 10X stocks of YU (38) or carbachol (Sigma–Aldrich) were made in FluoroBrite DMEM, and 100 μl was added directly to the dish at the 1-min mark during imaging. Time-lapse images were collected at 10 s intervals on an Olympus IX83 inverted microscope with a Lumencor Sola light engine and a 475 nm excitation/525 nm emission filter set using a 10X objective and a Hamamatsu ORCA-FLASH4.0 sCMOS camera. All images were collected at the full camera dynamic range using Micro-Manager software (71), and the same thresholds were used to process all images. A 25-pixel radius circle was used to collect fluorescent intensity data from each cell at each time point in FIJI (ImageJ) (72), which was then converted to change in fluorescence over initial fluorescence (ΔF/F_0_).

### Fluorescence microscopy

Cells were plated on MatTek dishes with coverglass bottoms coated with fibronectin and allowed to adhere overnight at 37 °C. Live-cell imaging was performed on a Nikon Ti2 inverted microscope equipped with a 60X oil immersion objective and a Yokogawa CSU-W1 spinning disk confocal attached to a Hamamatsu ORCA-FLASH4.0 sCMOS camera. Image stacks were captured at 16 bit 2048 × 2044 resolution with an axial spacing of 0.5 μm using the Nikon Elements Software package. All images were collected at the full camera dynamic range, and the same thresholds were used to process all images. Specific Z-slices were chosen for figure presentation that represented the z-midline of the cell body. Orthogonal reslicing was performed in FIJI. Line scans were performed in FIJI by selecting four pixel-wide rectangles and using the multiplot tool in the ROI manager plugin. Independent line scans were performed on 10 different cells for colocalization, and Pearson’s coefficients were determined for each scan. For PM localization, one line scan was performed for each cell ensuring that each line included cytoplasm between the PM and nucleus and was perpendicular to the PM. For FLAG imaging, cells were fixed in PBS with 4% paraformaldehyde and 0.2% glutaraldehyde for 10 min at room temperature, then permeabilized with 0.1% Triton X-100 in PBS for 5 min, washed with PBS, and blocked with 2% fish gelatin (Sigma–Aldrich) in PBS. Primary and secondary antibodies were diluted in 2% fish gelatin in PBS. Primary ant-FLAG M2 mouse monoclonal antibody (Sigma–Aldrich), and secondary goat anti-mouse Alexafluor-488 (Life Technologies) were used, and the mounting agent was ProLong Gold + 4′,6-diamidino-2-phenylindole (Life Technologies). Imaging was performed on an Olympus IX83 inverted microscope equipped with a 100X oil immersion objective and a Yokogawa CSU-W1 spinning disk confocal attached to an Andor Zyla-4.2 sCMOS camera.

### Palmitoylation and immunoprecipitation

HEK293 cells stably expressing 3XFLAG-INPP5A were labeled with 25 μM 17-ODYA for 6 h in DMEM (Invitrogen) supplemented with 5% FBS, 0.1 mm sodium pyruvate, and 0.1 mM nonessential amino acids in the presence or the absence of 20 μM HDFP. For pulse-chase experiments, 17-ODYA-treated cells were washed with PBS prior to being chased with media containing 200 μM palmitate for the indicated periods. At the end of each experiment, cells were washed and suspended in a lysis buffer (PBS supplemented with 2.5 μM PMSF, 1× EDTA-free Complete Protease Inhibitors, and 1% Triton X-100). Cleared lysates were immunoprecipitated overnight at 4 °C with mouse anti-FLAG M2-agarose for 2 h at 4 °C. After three washes with lysis buffer, beads were suspended in PBS. Click chemistry reaction protocols were adopted from previous publications (73, 74). Immunoprecipitated samples were reacted with click chemistry reagents (20 μmM TAMRA-azide, 1 mM Tris(2-carboxyethyl)phosphine hydrochloride, 100 μM Tris-(benzyltriazolylmethyl)amine, and 1 mM CuSO_4_) for 1 h at room temperature with periodic mixing. Reactions were stopped by addition of reducing SDS-PAGE sample buffer and boiling for 5 min at 100 °C. Samples were separated by SDS-PAGE and analyzed by in-gel fluorescence analysis (Typhoon 9400 laser scanner; GE Healthcare) and Western blotting. Bands of interest were quantified using ImageJ for TAMRA fluorescence. Western blot analyses were performed according to the following protocol. Samples were separated by SDS-PAGE, transferred to polyvinylidene difluoride (Millipore; catalog no.: IPVH00010), blocked for at least 1 h using 5% (w/v) milk in TBST (25 mM Tris (pH 7.2), 150 mM NaCl, 2.7 mM KCl, 0.1% (v/v) Tween-20), incubated with mouse anti-FLAG primary monoclonal antibody in blocking buffer for at least 2 h, washed three times with TBST, incubated for 1 h with appropriate horseradish peroxidase–conjugated secondary antibodies diluted in blocking buffer, and washed three times with TBST. Membranes were then incubated with IRDye 800 Goat anti-mouse (LI-COR). Following incubation, membranes were washed at least three times with TBST, and signals were detected using LI-COR Odyssey model 9120 imaging system (LI-COR). All quantified Western blot signals were within the linear range of the detection system as determined by an independent standard curve.

### Curated data and statistical analyses

Data from the Cancer Dependency Map Project were obtained through the DepMap Portal (https://depmap.org/portal/). Downloaded DepMap data were graphed in GraphPad Prism (version 10.3.1; GraphPad Software, Inc). Protein abundance data for MP41, MP46, and OCM-1A cell lines were calculated previously (27) and regraphed in GraphPad Prism. Data from the Cancer Genome Atlas Project (The Cancer Genome Atlas [TCGA]; https://www.cancer.gov/tcga) were collected using the cBioPortal Cancer Genomics webtool (https://www.cbioportal.org) using a combined set of the UM (TCGA Pan-Cancer Atlas: UVM, 80 samples) and SKCM (TCGA Pan-Cancer Atlas: 448 samples) samples and searching by Gene (*INPP5A*). The data were downloaded as text files and regraphed in GraphPad Prism. All statistical analyses were performed in GraphPad Prism (version 10.3.1). Average data values and error bars on graphs represent means with standard deviation, except for Figure 1, *F* and *G* (TCGA data), which represent median and interquartiles. Stars indicate significance as determined by statistical analysis (∗<0.05, ∗∗<0.01, and ∗∗∗<0.01). Data for treated and untreated samples were analyzed *via* unpaired *t* tests or Mann–Whitney tests. Nonlinear regression one-phase exponential decay was used for palmitoylation pulse-chase half-life estimation.

## Data availability

All processed data are contained within the article and in the supporting information. Original data files are available on request from the corresponding authors.

## Supporting information

This article contains supporting information.

## Conflict of interest

M. D. O. and K. J. B. are listed as coinventors on a provisional patent application on Targeted pharmacological therapeutics in uveal melanoma that is owned by Washington University in St Louis. All other authors declare that they have no conflicts of interest with the contents of this article.

## Supporting information

Supplemental_Figure_Legends

S1_HeLa_IP3R1_mCI5

S4_MP46_OGB1_FR900359

S3_MP46_OGB1

S5_OCM1A_OGB1

S5_OCM1A_OGB1

S6_MP46_OGB1_noCa

S7_FNAB_OGB1

S8_FNAB_OGB1_FR900359

S9_MP46_OGB1_YU144369

S10_HEK293_OGB1_carbachol

S11_Onken_Proteomics_Data_Reanalysis
