# Supplementary material for: Inositol (1,4,5)-trisphosphate 5-phosphatase promotes survival of uveal melanoma by regulating oncogenic G protein–driven calcium oscillations
Source: J Biol Chem. 2025 Aug 12;301(9):110589. doi: 10.1016/j.jbc.2025.110589 (PMC12450637; doi:10.1016/j.jbc.2025.110589)
Supplement: Supplemental_Figure_Legends [file mmc1.docx]

**Supplemental movie S1:** Live-cell fluorescence video microscopy showing coincidence of plasma membrane-localized mCherry-INPP5A (magenta) and immobile IP3 receptor (EGFP-ITPR1, yellow) clusters at junctions between the plasma membrane and ER. See figure 2D for more information.

**Supplemental movie S2:** Live-cell fluorescence video microscopy showing MP41 (GNA11-Q209L) UM cells loaded with Oregon green 488-BAPTA-1-AM. Fluorescence emission was measured over time to detect changes in cytoplasmic Ca2+ levels. See figure 4 for more information.

**Supplemental movie S3:** Live-cell fluorescence video microscopy showing MP46 (GNAQ-Q209L) UM cells loaded with Oregon green 488-BAPTA-1-AM. Fluorescence emission was measured over time to detect changes in cytoplasmic Ca2+ levels. See figure 4 for more information.

**Supplemental movie S4:** Live-cell fluorescence video microscopy showing MP46 (GNAQ-Q209L) UM cells loaded with Oregon green 488-BAPTA-1-AM after 24-hour treatment with the GNAQ/11 inhibitor FR900359. Fluorescence emission was measured over time to detect changes in cytoplasmic Ca2+ levels. See figure 4 for more information.

**Supplemental movie S5:** Live-cell fluorescence video microscopy showing OCM-1A (BRAF-V600E) UM cells loaded with Oregon green 488-BAPTA-1-AM. Fluorescence emission was measured over time to detect changes in cytoplasmic Ca2+ levels. See figure 4 for more information.

**Supplemental movie S6:** Live-cell fluorescence video microscopy showing MP46 (GNAQ-Q209L) UM cells loaded with Oregon green 488-BAPTA-1-AM and imaged in DPBS containing no Ca2+. Fluorescence emission was measured over time to detect changes in cytoplasmic Ca2+ levels. See figure 4 for more information.

**Supplemental movie S7:** Live-cell fluorescence video microscopy showing cells isolated from a primary human UM tumor loaded with Oregon green 488-BAPTA-1-AM. Fluorescence emission was measured over time to detect changes in cytoplasmic Ca2+ levels. See figure 5 for more information.

**Supplemental movie S8:** Live-cell fluorescence video microscopy showing cells isolated from a primary human UM tumor loaded with Oregon green 488-BAPTA-1-AM after 24-hour treatment with the GNAQ/11 inhibitor FR900359. Fluorescence emission was measured over time to detect changes in cytoplasmic Ca2+ levels. See figure 5 for more information.

**Supplemental movie S9:** Live-cell fluorescence video microscopy showing MP46 (GNAQ-Q209L) UM cells loaded with Oregon green 488-BAPTA-1-AM. At 1 minute after starting imaging (frame 6), the INPP5A inhibitor YU144369 was injected into the imaging chamber. Fluorescence emission was measured over time to detect changes in cytoplasmic Ca2+ levels. See figure 6 for more information.

**Supplemental movie S10:** Live-cell fluorescence video microscopy showing control HEK293 cells and HEK293 cells expressing INPP5A isoforms loaded with Oregon green 488-BAPTA-1-AM. At 1 minute after starting imaging (frame 6), the muscarinic cholinergic receptor agonist carbachol was injected into the imaging chamber (20 µM final concentration). Fluorescence emission was measured over time to detect changes in cytoplasmic Ca2+ levels. See figure 6 for more information.

**Supplemental data file S11:** Reanalyzed protein abundance data from prior proteomics experiments downloaded from the PRIDE repository (accession PXD038115) (22) for cross-comparison among cell lines.
